# Supplementary material for: 4,5-dihydroxyhexanoic acid is a robust circulating and urine marker of mitochondrial disease and its severity
Source: bioRxiv. 2026 Feb 12:2026.02.10.705117. Preprint. [Version 1] doi: 10.64898/2026.02.10.705117 (PMC12918946; doi:10.64898/2026.02.10.705117)
Supplement: Supplement 1 [file NIHPP2026.02.10.705117v1-supplement-1.pdf]

bioRxiv preprint doi: https://doi.org/10.64898/2026.02.10.705117; this version posted February 12, 2026. The copyright holder for this preprint (which was not certified by peer review) is the author/funder, who has granted bioRxiv a license to display the preprint in perpetuity. It is made available under aCC-BY 4.0 International license.

Supplementary Information

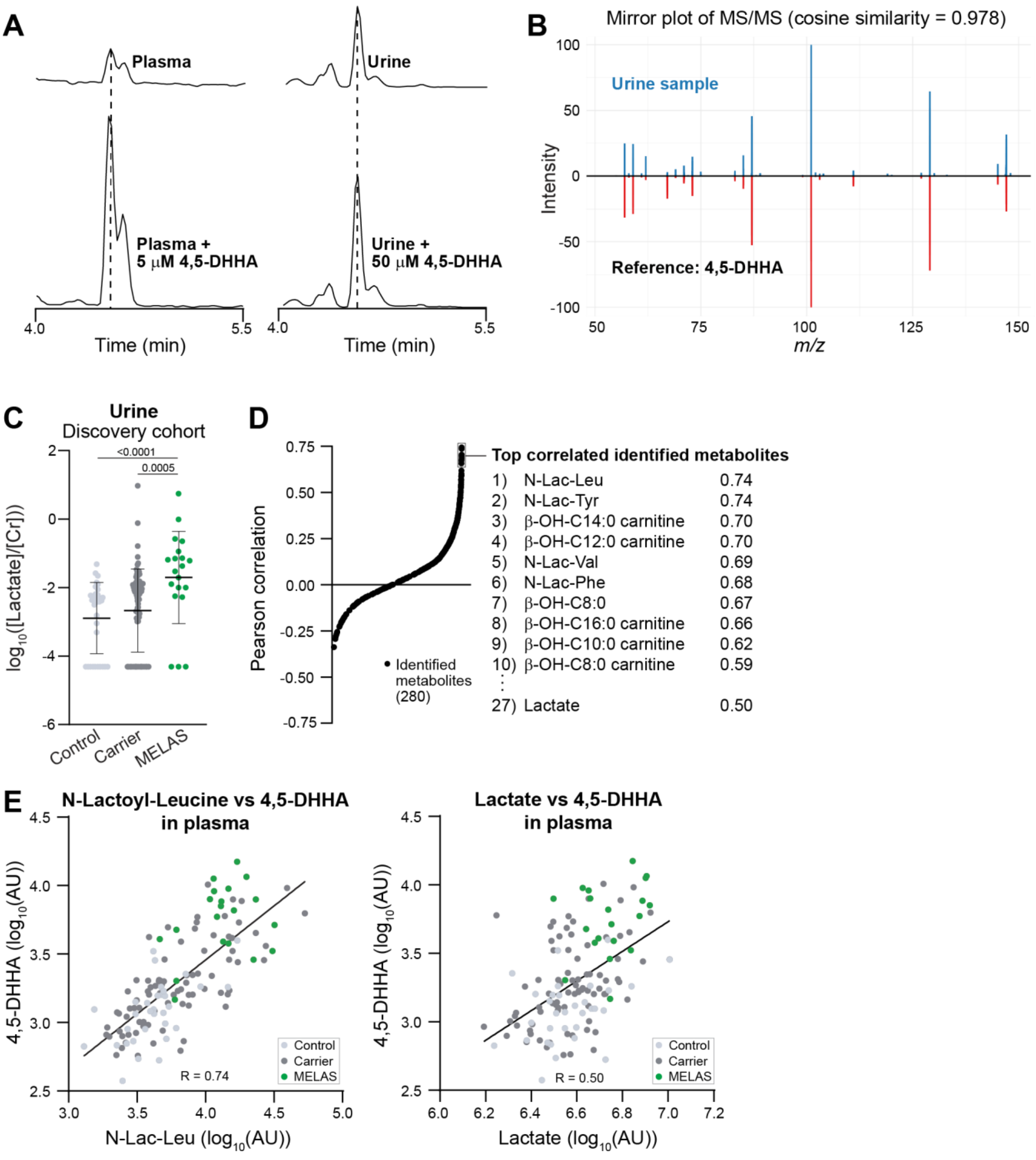

Fig. S1. A) Extracted ion chromatograms for U147 (147.0662 *m/z*) have the same retention time as each other and as a chemically synthesized pure standard of 4,5-DHHA. **B)** Mirror plot of sample and synthesized 4,5-DHHA shows high cosine similarity score. **C)** Urine lactate/Cr is

bioRxiv preprint doi: https://doi.org/10.64898/2026.02.10.705117; this version posted February 12, 2026. The copyright holder for this preprint (which was not certified by peer review) is the author/funder, who has granted bioRxiv a license to display the preprint in perpetuity. It is made available under aCC-BY 4.0 International license.

significantly elevated in MELAS patients compared to Controls and Carriers (lactate concentrations of zero were set to one-tenth the minimum lactate concentration and p-values are based on Mann-Whitney U test). **D-E)** Correlation of 4,5-DHHA with 280 identified metabolites across the Discovery cohort shows it is most strongly correlated with top markers like N-lactoyl-leucine and only moderately correlated with plasma lactate.

bioRxiv preprint doi: https://doi.org/10.64898/2026.02.10.705117; this version posted February 12, 2026. The copyright holder for this preprint (which was not certified by peer review) is the author/funder, who has granted bioRxiv a license to display the preprint in perpetuity. It is made available under aCC-BY 4.0 International license.

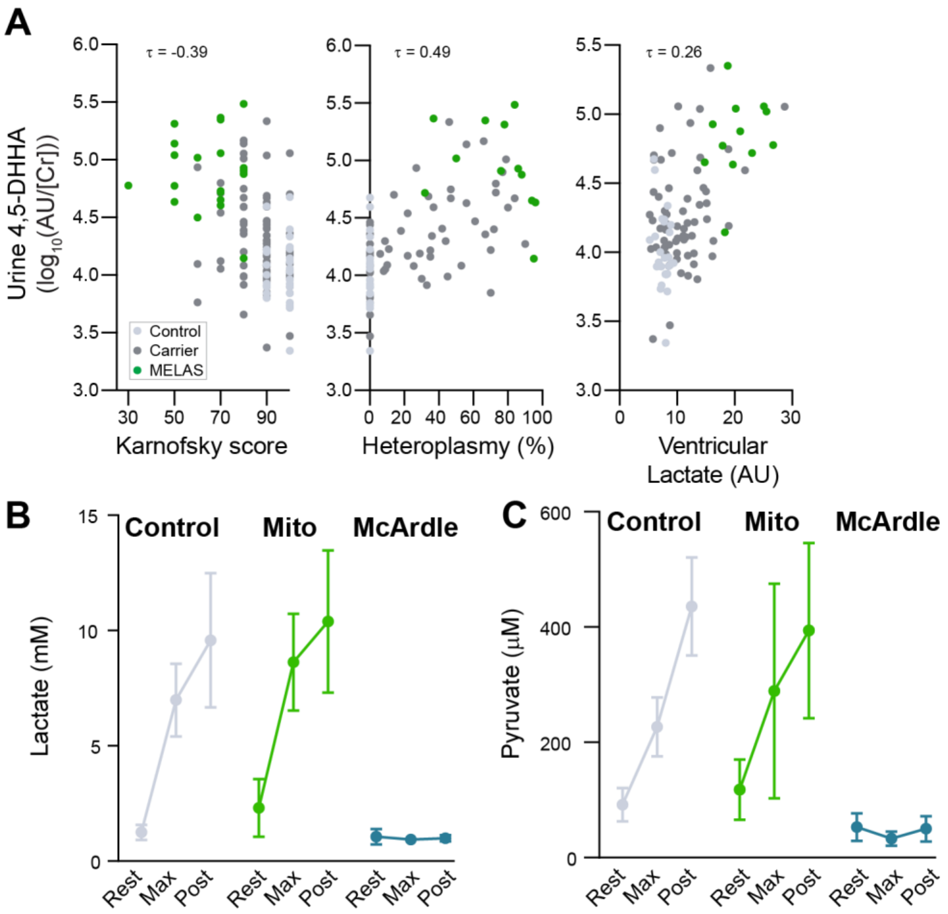

Fig. S2. A) Urine 4,5-DHHA is strongly correlated with three metrics of disease severity – Karnofsky performance score, urine heteroplasmy, and ventricular lactate. **B, C)** Plasma lactate and pyruvate levels rise during exercise in healthy controls and patients with mitochondrial myopathy while their levels remain low in McArdle disease.
